# The Possible Hematological Cost of Metabolic Success: Do Incretin-Based Therapies Silently Trigger Anemia?

**DOI:** 10.3390/medsci14050577

**Published:** 2026-09-17

**Authors:** Maja Mizdrak, Darko Batistić, Ivan Mizdrak

**Affiliations:** 1 Department of Internal Medicine, University Hospital of Split, Split School of Medicine, 21000 Split, Croatia; 2 Department of Ophthalmology, University Hospital of Split, Split School of Medicine, 21000 Split, Croatia; 3 Department of ENT, University Hospital of Split, Split School of Medicine, 21000 Split, Croatia

**Keywords:** anemia, incretin-based therapy, GLP-1, GIP, gliptins, malnutrition, obesity, diabetes mellitus, iron, micronutrients, hepcidin

## Abstract

Diabetes mellitus and obesity represent a growing global health burden with high morbidity and mortality. In the last several years, novel incretin-based therapies have been developed, and their use has risen dramatically. Beyond their proven metabolic, glycemic, cardiovascular, renal, and many other benefits, there are some observational data linking anemia development with their use. From the era of gliptins to retatrutide, in this review we tried to explore whether erythroid suppression is the hidden paradigm of advanced incretin-based therapies. Potential pathophysiological explanations include micronutrient vulnerabilities due to reduced food intake, lower dietary diversity, gastrointestinal intolerance, delayed gastric emptying, and weight loss. The most important concerns involve deficiencies in iron, vitamin B12, vitamin B2, vitamin D, calcium, magnesium, and zinc, along with others such as thiamine, folate, vitamin A, and potassium. The available evidence indicates a complex and heterogeneous relationship between incretin-based therapies and hematological parameters, with findings differing across therapies, study populations, and outcomes. Although observational studies suggest possible associations with anemia, iron deficiency, and nutritional deficiencies in selected populations, current evidence is insufficient to establish a direct causal relationship or a class-wide adverse effect. Further large-scale studies are needed, as well as increased clinician awareness of potential hematological and nutritional consequences that require laboratory follow-up before and during medication prescription.

## 1. Introduction

Incretins are gut-derived hormones and members of the glucagon superfamily that are released from the small intestine after food intake [1]. From a physiological viewpoint, glucagon-like peptide-1 (GLP-1) and glucose-dependent insulinotropic peptide (GIP) are the primary incretins, and treatments based on incretins, including DPP-4 inhibitors, GLP-1 receptor agonists, and combined GIP/GLP-1 receptor agonists, utilize gut hormone pathways to manage diabetes mellitus and obesity [2]. Type 2 diabetes mellitus (T2DM) is a complex disease often accompanied by obesity, fatty liver, osteoporosis, chronic kidney disease, and other metabolic disorders, making treatment even more challenging [3]. According to statistics from 2024, approximately 11% of adults worldwide have diabetes, while 42% are affected by obesity [4]. Alarmingly, around 252 million adults remain undiagnosed with diabetes, and more than 3.4 million deaths—accounting for 9.3% of global mortality—are linked to the condition [4]. Despite projections of a 25% increase in the global population over the next 25 years, diabetes and obesity rates are expected to rise by 45% [4]. Beyond glucose regulation and weight loss, incretin-based therapies have shown notable improvements in overall cardiorenal–metabolic health. Consequently, their use has increased significantly. For instance, in 2023, U.S. prescriptions included 25,954,067 for semaglutide, 10,358,270 for dulaglutide, 6,310,174 for tirzepatide, and 2,191,691 for liraglutide [5]. In Europe, prescriptions rose by 24% compared to numbers from 2021 [6]. The primary side effects of these drugs include mild gastrointestinal issues, such as nausea, vomiting, diarrhea, and constipation, while pancreatitis remains rare [7]. However, emerging clinical data suggest a potential association between incretin-based therapy and hematological changes, including anemia in selected populations. Anemia can worsen fatigue, impair quality of life, reduce tissue oxygenation levels, and consequently exacerbate renal, metabolic, and cardiovascular conditions. Additionally, it is not identified as a common adverse reaction in the current product information for representative GLP-1 receptor agonists; however, it should be noted that safety labeling should be evaluated separately for each individual agent [8,9]. This narrative review aims to critically evaluate the available evidence regarding the potential association between incretin-based therapies and hematological changes.

## 2. Materials and Methods

This narrative review was conducted using a structured literature search of PubMed/MEDLINE, Scopus, and Google Scholar. The search covered publications available from database inception through 31 July 2026, with particular emphasis on studies published from 2022 onward to capture recent evidence regarding contemporary incretin-based therapies. Search terms were developed around the main concepts of incretin-based therapy, anemia, hematological parameters, iron metabolism, micronutrient deficiency, and erythropoiesis, and included combinations of the following terms: “anemia”, “incretins”, “GLP-1”, “GLP1-RA”, “GIP”, “DPP-4 inhibitors”, “gliptins”, “hepcidin”, “semaglutide”, “tirzepatide”, “dulaglutide”, “liraglutide”, “retatrutide”, “iron deficiency”, “diabetes mellitus”, “obesity”, “malnutrition”, and “micronutrients”. In PubMed/MEDLINE, terms were combined using Boolean operators (AND/OR) and applied to titles, abstracts, and relevant indexing terms. Equivalent combinations of keywords were adapted to the search interfaces and indexing systems of Scopus and Google Scholar. The search was supplemented by screening the reference lists of relevant articles and reviews to identify additional eligible studies.

Studies were considered eligible if they investigated the association between incretin-based therapies and anemia, hemoglobin or hematocrit changes, erythropoiesis, iron metabolism, or nutritional deficiencies potentially relevant to hematological outcomes. Clinical trials, prospective and retrospective cohort studies, observational studies, case reports, case series, mechanistic studies, in vitro and animal studies, and relevant high-quality reviews were considered. Duplicate publications were removed, and when studies involved overlapping populations, the publication providing the most relevant or comprehensive data was preferentially considered, while additional publications were retained when they provided distinct or complementary information. Study screening was performed independently by two authors through title and abstract review, followed by full-text assessment, with disagreements resolved by discussion and consensus. No language restrictions were applied. Preclinical studies were included primarily to assess biological plausibility and potential mechanisms, whereas case reports and case series were considered signals of potential associations and were not used to establish causality or estimate anemia incidence. Hematological outcomes were evaluated based on changes in hemoglobin, hematocrit, erythrocyte count, mean corpuscular volume, and iron-related parameters, including ferritin, transferrin saturation, and hepcidin, where available. Given the heterogeneity of the included studies, no uniform definition of anemia was retrospectively applied. When a formal diagnosis of anemia was reported, the diagnostic criteria or hemoglobin threshold used in the original study were retained. Studies reporting changes in hemoglobin or hematocrit without a formal diagnosis of anemia were considered separately and were not classified as anemia outcomes. Similarly, iron-deficiency anemia was distinguished from anemia of unspecified or other etiologies whenever this information was available. This approach was used to preserve the original study-specific outcome definitions and to account for differences between randomized clinical trials and real-world observational studies. As this was a narrative review, no formal risk-of-bias assessment or quantitative meta-analytic synthesis was performed; however, findings were interpreted according to study design, population characteristics, outcome definitions, and methodological limitations, and different levels of evidence were not considered equivalent.

## 3. The Incretin System, Bone Metabolism, and Hematopoiesis

Incretins carry out their biological functions by binding to specific G protein-coupled receptors, primarily the GLP-1 and GIP receptors [1]. When incretins bind to these receptors on pancreatic β-cells, they trigger signaling pathways via adenylate cyclase, leading to increased intracellular levels of cyclic adenosine monophosphate (cAMP) [1]. However, both GLP-1 and GIP are rapidly inactivated—lasting only 1 to 2 min in circulation—due to enzymatic cleavage driven by dipeptidyl peptidase-4 (DPP-4) [10]. DPP-4 is a serine protease that cleaves peptides with selective amino acids at the penultimate N-terminal position and is expressed on various cell surfaces (vascular endothelial cells, renal cells, and T lymphocytes) [10,11], and in the gut, three primary cell types display DPP-4 (CD26) expression and activity: enterocytes, hematopoietic cells, and vascular endothelial cells [11]. DPP-4 substrates include several chemokines, colony-stimulating factors, and interleukins [12,13]. In addition to its membrane-bound form, DPP-4 also exists as a soluble enzyme in plasma [10], and even without its transmembrane domain, this soluble form remains enzymatically active, interacting broadly with peptide substrates across multiple organs, including the gut, liver, lungs, and kidneys [10,11]. Thus, GLP-1 receptor agonists (GLP-1RAs) are engineered to resist DPP-4 degradation, thereby extending their biological half-life and therapeutic effects [1,2]. In contrast, DPP-4 inhibitors prevent the degradation of endogenous GIP and GLP-1, resulting in a modest increase in their physiological circulating levels (picomolar range), which remain significantly lower than the therapeutic concentrations achieved with synthetic GLP-1 analogs (nanomolar range) [1]. Although the incretin system is predominantly recognized for its role in glucose homeostasis and insulin secretion, recent research highlights its pleiotropic actions across various physiological processes beyond glycemic control, including immune modulation, vascular protection, improved endothelial function, blood pressure regulation, reductions in arterial stiffness and atherosclerosis, attenuation of oxidative stress, anti-fibrotic activity, and renal protection [1,14,15].

DPP-4 is expressed on all subsets of hematopoietic stem cells [12,13], and its function within the bone marrow microenvironment is dual-acting, functioning as both a receptor and an enzyme. It also participates in the regulation of hematopoietic stem cell trafficking and several hematopoietically relevant signaling pathways [12,13]. Stromal cell-derived factor 1 (SDF-1, also known as CXCL12) is constitutively produced in the bone marrow, playing a central role in stem cell anchoring and retention [13,16]; however, CD26/DPP-4 cleavage disrupts this signaling axis. Thus, experimental studies suggest that DPP-4 inhibition may enhance hematopoietic stem cell mobilization and recovery and may act synergistically with erythropoietin [13,17]. DPP-4 can cleave and modify the activity of several hematopoietically relevant cytokines and growth factors, including granulocyte–macrophage colony-stimulating factor (GM-CSF), interleukin-3 (IL-3), and erythropoietin (EPO) [13]. However, the potential consequences of this activity for hematopoiesis during pharmacological DPP-4 inhibition remain incompletely understood [13].

GLP-1 receptors are expressed on bone marrow stromal cells and adipocytes but not directly on mature osteoblasts [18,19,20]. Thus, GLP-1 receptor activation promotes stem cell differentiation toward the osteogenic lineage while inhibiting adipogenic differentiation by modulating the PKA/β-catenin and PKA/PI3K/AKT/GSK3β signaling pathways [18,19]. This increases osteoblast density and circulating bone formation marker levels while reducing marrow adipose tissue accumulation, providing a molecular rationale for the therapeutic potential of GLP-1 in osteoporosis [18,19]. Conversely, GIP receptors are expressed directly on osteoblasts, osteoclasts, and bone marrow mesenchymal stem cells [20,21]. The direct binding of GIP to its receptor stimulates osteogenesis and suppresses bone resorption [21]. Consequently, incretin hormones significantly modulate bone metabolism and the bone marrow niche, although their direct actions—particularly those of GLP-1 and GIP on hematopoietic stem cells—remain to be fully characterized. Mantelmacher et al. demonstrated that GIP plays an essential role in bone marrow hematopoiesis [22]. In their study, Gipr (−/−) mice exhibited a significant reduction in bone marrow hematopoietic stem/progenitor cells and myeloid progenitors, accompanied by a decline in both bone marrow and circulating immune cells [22]. The authors concluded that intact GIP signaling is essential for establishing supportive hematopoietic niches during reconstitution in radioablated bone marrow chimeras [22]. Interestingly, CXCL12 expression was upregulated in whole bone marrow extracts from Gipr (−/−) mice [22]. Together, these findings underscore the complex pathophysiological interplay in obesity, which is characterized by excessive white adipose tissue accumulation and systemic low-grade inflammation [3]. This state can shift bone marrow mesenchymal stem cell fate toward adipogenesis over osteogenesis, depleting osteoblast lineages and expanding bone marrow adiposity [3]. However, the precise pathophysiological mechanisms underlying these lineage shifts and their hematological ramifications remain incompletely understood, warranting further investigation.

## 4. DPP4 Inhibitors and Anemia: Data from In Vitro and In Vivo Studies

### 4.1. Mechanistic and In Vitro Evidence

Experimental in vitro studies have demonstrated that DPP-4 inhibition can enhance the activity of selected colony-stimulating factors in hematopoietic progenitor cells [13], providing biological plausibility for a potential hematopoietic effect of DPP-4 inhibition. However, these experiments primarily involved isolated cells, recombinant factors, or experimental DPP-4 inhibition and therefore could not establish that clinically used gliptins exert the same effects in humans. Other in vitro studies have investigated the effects of DPP-4 inhibition on cellular proliferation, migration, apoptosis, and tumor-cell behavior [23,24,25,26,27]. These findings indicate that DPP-4 may have context-dependent effects on cellular function. Importantly, some experimental studies reporting cytotoxic effects used drug concentrations several orders of magnitude higher than those achievable during routine clinical treatment [28]. Therefore, such findings should be interpreted as mechanistic or preclinical evidence rather than as evidence of a clinically established hematological effect.

### 4.2. Animal Studies

Animal studies provide additional evidence that DPP-4 inhibition may influence hematopoietic recovery. Broxmeyer et al. demonstrated that hematopoiesis following radiation or chemotherapy was enhanced in Dpp4-deficient mice and in mice treated with a DPP-4 inhibitor [13]. These findings suggest that DPP-4 inhibition may enhance the activity of DPP-4-sensitive hematopoietic factors and facilitate the recovery of hematopoietic progenitor cells following bone marrow injury [13]. Although these findings provide important insights into potential biological mechanisms, they cannot be automatically translated to humans. In particular, the experimental enhancement of cytokine activity should not be equated with a clinical increase in hemoglobin concentrations.

### 4.3. Human Observational Studies

Human evidence concerning the relationship between DPP-4 inhibitors and anemia remains limited. Data from a Japanese cohort of patients receiving hemodialysis showed that DPP-4 inhibitor treatment was associated with an improvement in the erythropoietin resistance index among patients without iron deficiency [29]. This finding suggests a possible association between DPP-4 inhibition and erythropoietic responsiveness in a selected population with chronic kidney disease. Zeng et al. retrospectively evaluated 443 patients with diabetic kidney disease who initiated DPP-4 inhibitor therapy and assessed hemoglobin concentrations before and after treatment [23]. They found that hemoglobin levels decreased slightly from 119.8 ± 20.7 to 118.7 ± 21.2 g/L; however, this decline was smaller than that observed before treatment initiation [23]. The authors therefore suggested that DPP-4 inhibitors may attenuate hemoglobin decline in diabetic kidney disease. A study of 110 adults with diabetes mellitus without major confounding comorbidities found no significant differences in hemoglobin or mean corpuscular volume between patients receiving metformin alone and those receiving metformin plus a DPP-4 inhibitor after 8 months of follow-up [30]. Additionally, in a cohort of 28,441 patients with T2DM, the cumulative 5-year incidence rate of anemia was lower among SGLT2 inhibitor users than among DPP-4 inhibitor users (6.9% vs. 11.3%) [31]. Another cohort study reported higher hemoglobin concentrations among SGLT2 inhibitor users compared with DPP-4 inhibitor users [32]. These studies, however, were not designed to demonstrate a direct hematopoietic effect of DPP-4 inhibition.

### 4.4. Clinical Trial Evidence

Clinical trial evidence specifically addressing anemia or hemoglobin outcomes during DPP-4 inhibitor therapy remains limited. A randomized phase II trial evaluated sitagliptin for the prevention of acute graft-versus-host disease in patients undergoing hematopoietic stem cell transplantation [33], and although the study supports the potential clinical relevance of DPP-4 inhibition in the hematopoietic and immune setting, its primary outcome was graft-versus-host disease rather than anemia or hemoglobin concentration. Importantly, available clinical studies have not directly demonstrated that DPP-4 inhibitors preserve circulating GM-CSF, G-CSF, IL-3, or EPO levels in humans at concentrations corresponding to routine clinical treatment. Thus, the preservation of hematopoietically active DPP-4 substrates should currently be regarded primarily as a mechanistic and preclinical hypothesis rather than an established clinical mechanism.

### 4.5. Interpretation and Limitations

Overall, the evidence linking DPP-4 inhibition with anemia remains heterogeneous. Mechanistic and in vitro studies demonstrate that DPP-4 can modify the activity of several hematopoietically relevant substrates, while animal studies indicate that DPP-4 inhibition may enhance hematopoietic recovery after bone marrow injury [13]. Human observational studies suggest possible effects on erythropoietin responsiveness or the trajectory of hemoglobin concentrations in selected populations [23,29]. However, these findings do not establish a direct causal effect of DPP-4 inhibitors on erythropoiesis or anemia. Thus, prospective clinical studies incorporating predefined hematological endpoints, iron-status parameters, and erythropoietic markers are needed to determine the clinical significance of this pathway.

## 5. GLP-1 Receptor Agonists and Hematological Outcomes: Risk Factors and Indirect Mechanisms

### 5.1. Metabolic Scope and Hematological Divergence

Glucagon-like peptide-1 receptor agonists regulate energy balance and glucose homeostasis by binding to GLP-1 receptors. Their physiological actions include glucose-dependent insulin secretion, postprandial glucagon suppression, delayed gastric motility, and central appetite reduction. Beyond glycemic control, prospective cardiovascular and renal outcome trials, such as SUSTAIN-6, PIONEER, and the FLOW trial, have demonstrated cardiovascular, metabolic, and renal benefits [34,35,36]. Other therapeutic implications include arterial hypertension, systemic anti-inflammatory signaling, insulin sensitization, polycystic ovary syndrome (recently renamed Polyendocrine Metabolic Ovarian Syndrome), metabolic dysfunction-associated steatotic liver disease, and obstructive sleep apnea [37,38,39,40,41,42,43]. However, their direct hematoprotective capacity remains far less well characterized.

### 5.2. Bone Marrow, Renal, and Hepatic Pathways

Preclinical models indicate that GLP-1 receptor activation shifts osteo-adipogenic differentiation toward osteoblastogenesis and mitigates oxidative stress, preserving the structural integrity of the bone marrow hematopoietic microenvironment [13,44]. Clinically, renal preservation represents a major indirect erythropoietic pathway in diabetic nephropathy [45]. By modulating intrarenal hemodynamics and reducing glomerular hyperfiltration, GLP-1 and GIP receptor signaling may preserve the peritubular capillary architecture and interstitial cells responsible for endogenous erythropoietin synthesis. Under acute ischemic or hypoxic strain, GLP-1 receptor stimulation blunts tubular cell apoptosis and stabilizes hypoxia-inducible factor-1 alpha [18,46]. Additionally, this renoprotective mechanism is supported by clinical outcome trials. In the landmark FLOW trial evaluating 3533 patients with type 2 diabetes and chronic kidney disease, semaglutide significantly attenuated eGFR decline and reduced major renal events [36]. Renal preservation could theoretically influence anemia risk in patients with diabetic kidney disease because progressive loss of kidney function is associated with impaired erythropoietin production. However, whether the renal benefits observed with GLP-1 receptor agonists translate into the preservation of endogenous erythropoietin production has not been directly demonstrated. In the FLOW trial, semaglutide slowed kidney function decline and reduced major kidney outcomes in patients with type 2 diabetes and chronic kidney disease, but the trial was not designed to establish an erythropoietic mechanism. Conversely, in experimental models of hepatic ischemia–reperfusion injury, liraglutide was associated with reduced hepatic iron accumulation and altered hepcidin-related signaling; however, the relevance of these findings to systemic iron homeostasis or anemia during clinical GLP-1 receptor agonist therapy remains uncertain [47].

### 5.3. The Hepcidin–Ferroportin Axis in Incretin Therapy

Hepcidin is the principal regulator of systemic iron homeostasis. It binds to the cellular iron exporter ferroportin, promoting its internalization and degradation and thereby reducing iron export from duodenal enterocytes, hepatocytes, and reticuloendothelial macrophages [48,49,50]. Increased hepcidin activity can consequently restrict circulating iron availability and contribute to functional iron deficiency [48,49]. Thus, incretin therapies could theoretically influence this axis indirectly through changes in systemic inflammation, adiposity, and metabolic status; however, the direct modulation of the hepcidin–ferroportin pathway using incretin therapy has not been firmly established in humans. Weight loss, reductions in visceral adiposity, and improvements in metabolic status may attenuate inflammatory signaling, including interleukin-6-mediated activation of hepatic STAT3 pathways involved in hepcidin regulation [51,52], and such changes could potentially affect iron sequestration and systemic iron availability. However, while experimental and clinical observations indicate biological plausibility for a potential interaction between incretin therapy, inflammation, and iron metabolism, the available evidence remains limited and heterogeneous. In particular, changes in inflammatory markers, ferritin expression, transferrin saturation, or erythropoietin requirements should not be interpreted as direct evidence of altered hepcidin or ferroportin activity unless these pathways are specifically measured. Therefore, the hepcidin–ferroportin axis should currently be regarded as a potential indirect mechanistic pathway requiring further investigation rather than an established hematological effect of incretin-based therapy.

### 5.4. The Anemia Paradox: Clinical Evidence vs. Molecular Theory

**Figure 1:**
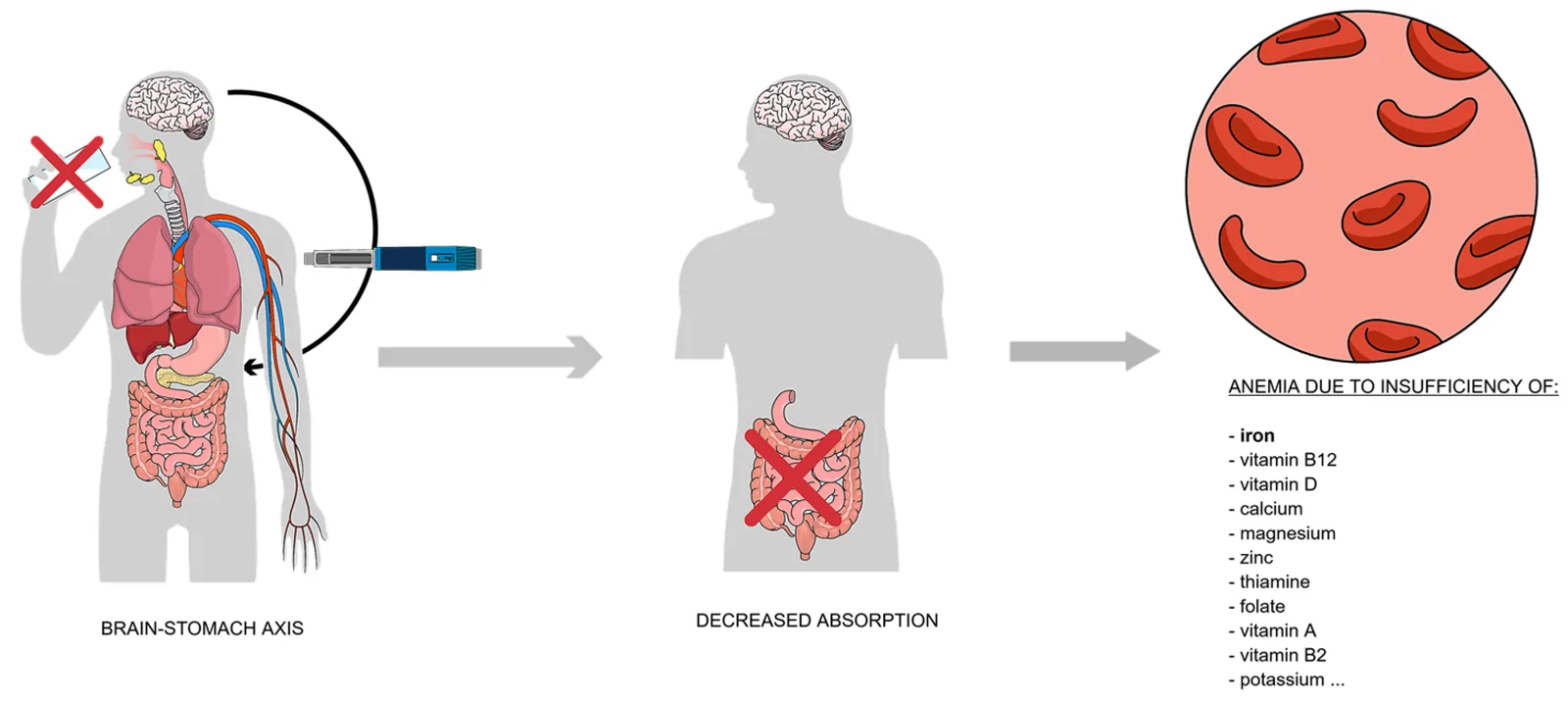
Proposed pathways that may contribute to hematological and nutritional changes during incretin-based therapy. The figure summarizes hypothesized mechanisms derived from preclinical, observational, and clinical evidence and does not imply established causality (original figure created by the authors for this manuscript according to data from references [49,50,51,52,53,54,55,56,57]).

The apparent ‘anemia paradox’ may reflect a discrepancy between the anti-inflammatory effects of incretin-based therapies and the hematological outcomes observed in clinical studies. Inflammatory signaling, particularly through the IL-6/hepcidin axis, is an established regulator of iron homeostasis, and attenuation of systemic inflammation could theoretically improve iron availability for erythropoiesis. However, whether GLP-1 receptor agonists directly modulate hepcidin and iron mobilization in humans remains insufficiently established. Therefore, the available clinical evidence does not support a consistent increase in hemoglobin concentrations following GLP-1 RA treatment. Instead, observational data suggest that nutritional deficiencies and iron-deficiency anemia may occur in a subset of patients, although these associations do not establish a direct causal relationship with incretin therapy. In a large retrospective cohort of 461,382 adults newly prescribed GLP-1 receptor agonists, Butsch et al. reported newly recorded nutritional anemia in 2.1% and 4.0% of patients at 6 and 12 months, respectively, including iron-deficiency anemia in 1.6% and 3.2% and other nutritional anemia types in 0.5% and 1.1% at the corresponding time points [53]. Overall nutritional deficiencies or deficiency-related complications were recorded in 12.7% of patients at 6 months and 22.4% at 12 months [53]. These findings indicate an association between GLP-1 receptor agonist use and subsequently recorded nutritional deficiencies and anemia diagnoses in this observational dataset; however, they do not establish a causal relationship between GLP-1 receptor agonist therapy and anemia. These findings may partly reflect reduced caloric intake and decreased dietary diversity during treatment, particularly among individuals with pre-existing micronutrient inadequacies (Figure 1) [54,55]. Additionally, vitamin D deficiency was reported in 7.5% and 13.6% of patients at 6 and 12 months, respectively [53]. Observational data suggest that different hematological and nutritional outcomes may occur in selected populations. Thus, these outcomes should not be considered interchangeable, as the available studies differ substantially in how these endpoints were defined and measured.

Several factors may contribute to the apparent discrepancy between the potential anti-inflammatory effects of incretin therapy and hematological outcomes. Gastrointestinal adverse effects, delayed gastric emptying, appetite suppression, and reduced food intake may decrease the intake of nutrients required for erythropoiesis, including iron, vitamin B12, and folate [55,58]. Emerging evidence also suggests that changes in iron absorption may occur during GLP-1 RA treatment. In addition, changes in hydration status and plasma volume may influence hemoglobin and hematocrit measurements independently of changes in total red blood cell mass [59]. Importantly, underlying comorbidities such as type 2 diabetes and chronic kidney disease may independently contribute to anemia, iron dysregulation, and impaired erythropoiesis, thereby complicating the attribution of hematological changes specifically to incretin therapy. The distinction between GLP-1 RAs and DPP-4 inhibitors should also be considered. Unlike DPP-4 inhibitors, GLP-1 RAs do not inhibit DPP-4 enzymatic activity and therefore do not exert their pharmacological effects by prolonging the half-life of endogenous DPP-4 substrates [54]. Importantly, the clinical relevance of this distinction to erythropoiesis and hemoglobin concentrations remains uncertain. Overall, the available evidence suggests that the apparent “anemia paradox” is likely multifactorial rather than attributable to a single mechanism. Although the anti-inflammatory actions of incretin-based therapies could theoretically favor improved iron availability, reduced dietary intake, gastrointestinal effects, nutritional vulnerability, and underlying metabolic or renal disease may counterbalance or obscure any potential hematological benefit.

### 5.5. Gastrointestinal and Microbiome Mechanisms

Nutritional deficiencies should be considered according to the strength and relevance of the available evidence. Iron, vitamin B12, folate, and riboflavin deficiencies are well-established causes of hematological abnormalities and may contribute to anemia when clinically significant. Some nutritional deficiencies have also been reported in patients receiving GLP-1-based therapies, although the extent to which incretin treatment directly contributes to these abnormalities remains uncertain. Other deficiencies, including those involving vitamin D, calcium, magnesium, zinc, thiamine, vitamin A, and potassium, may theoretically occur in the context of reduced dietary intake, gastrointestinal intolerance, or substantial weight loss, but their relationship with incretin-based therapy and hematological outcomes has not been adequately studied. Therefore, these nutritional factors should not be considered established hematological consequences of incretin therapy in the absence of direct clinical evidence. Several gastrointestinal mechanisms have been proposed as potential contributors to changes in iron availability during incretin-based therapy [58]. Delayed gastric emptying and altered upper gastrointestinal transit may interfere with iron solubilization and duodenal absorption [49,50,51,52,53,54,55,56,57,58,59]. Prolonged satiety and food aversion may lead to monotonous diets [60,61] and reduced consumption of iron-dense foods [62]. Although gastric acidity contributes to the solubilization and absorption of non-heme iron, whether incretin-based therapies produce clinically meaningful alterations in this process remains insufficiently established [63,64,65,66].

Secondary riboflavin (vitamin B2) deficiency can also develop as a result of low dietary intake or malabsorption [67]. Specifically, riboflavin deficiency can impair iron metabolism and hematopoietic function, but evidence directly linking clinically significant riboflavin deficiency to incretin therapy remains limited [68,69,70,71]. In parallel, insufficient protein and calcium intake may contribute to lean mass loss, while thiamine and cobalamin deficits may accumulate over time [55]. Finally, incretins may alter the gut microbiota, which modulates intestinal epithelial health, mucosal permeability, bile acid metabolism, and short-chain fatty acid synthesis [71,72]. Microbial metabolites stimulate GLP-1 release, while GLP-1 therapy influences gut inflammation and microbial diversity [71,72]. Indeed, baseline gut microbiome composition has emerged as a potential predictor of individual metabolic and gastrointestinal responses to semaglutide therapy [73].

### 5.6. Pharmacological vs. Weight Loss and Nutritional Drivers of Hematological Changes

Hematological changes observed during GLP-1-based therapies may reflect an interplay between potential direct pharmacodynamic effects and indirect metabolic adaptations. GLP-1 RAs may exert systemic anti-inflammatory effects that could influence circulating IL-6 and TNF-α levels and, potentially, hepatic hepcidin signaling and iron mobilization [57]. Conversely, delayed gastric emptying and alterations in intraluminal pH may affect the reduction and intestinal absorption of dietary iron [74]. Additionally, marked appetite suppression may restrict overall caloric and micronutrient intake, particularly heme iron, vitamin B12, and folate [56]. Substantial weight loss may alter dietary intake, nutritional status, and body-fluid distribution, potentially influencing hematological measurements; however, the extent to which these changes contribute to clinically relevant anemia during incretin therapy remains uncertain [56,75]. Furthermore, improved glycemic control may alter intravascular fluid dynamics, with acute hemodilution potentially lowering measured hemoglobin concentrations without compromising total erythrocyte mass [75,76]. Consequently, while systemic anti-inflammatory signaling could theoretically favor iron bioavailability, localized gastrointestinal effects, reduced nutrient intake, and substantial weight loss may represent countervailing factors that could influence circulating hemoglobin levels.

## 6. GLP-1 Receptor Agonists and Hematological Outcomes: Real-World Clinical Evidence

### 6.1. Comparative Anemia and Hematological Trends

Some observational studies highlight distinct hematological outcomes among newer antidiabetic classes. In a major comparative cohort, Lund et al. tracked 132,914 patients initiating SGLT2 inhibitors, GLP-1 receptor agonists, or DPP-4 inhibitors [76]. They found that SGLT2i users exhibited a significantly higher rate of secondary polycythemia than those in the GLP-1 RA and DPP-4i groups [76]. Conversely, DPP-4i initiation was associated with a higher risk of anemia compared with both SGLT2i and GLP-1 RA users [76]. Interestingly, GLP-1 RA initiators showed an early elevation in uterine cancer risk relative to SGLT2i and DPP-4i users, presenting a potential gynecologic source of blood loss [76]. In a cohort of 700 patients, Almuammar et al. noted a median hemoglobin decline of 2 g/L following GLP-1 analog initiation, while 8.4% of participants met the study definition of incident anemia [56]. Ferritin concentrations remained largely unchanged, while higher baseline hemoglobin expression was strongly associated with a lower likelihood of subsequent anemia onset [56]. In a pilot study of 51 adults with type 2 diabetes, semaglutide treatment for 10 weeks was associated with attenuated increases in serum iron, transferrin saturation, and ferritin following oral iron administration, corresponding to a median 13% reduction in iron absorption. However, the study was small, short-term, and exploratory and did not establish clinically significant iron deficiency or anemia [74]. This study assessed iron absorption and short-term biochemical responses to an oral iron challenge and did not establish biochemical iron deficiency, iron-deficiency anemia, or a clinically significant reduction in hemoglobin. Similarly, a multicenter retrospective study of 13,799 patients with diabetes and early chronic kidney disease (CKD stages 1–3) by Hu et al. found that SGLT2i users developed significantly fewer composite anemia events over a 2.5-year follow-up period than GLP-1 RA users [75]. Comparative observational studies have reported differences in anemia rates between GLP-1 RA and SGLT2 inhibitor users; however, these findings do not establish a causal effect of GLP-1 RAs on anemia. Because of the observational design, residual confounding and indication bias cannot be excluded. To better account for potential differences between treatment groups, recent large-scale real-world studies have employed propensity-score matching and multivariable regression models adjusting for baseline eGFR, concomitant medications, and baseline iron parameters. Furthermore, cardiovascular and renal outcome trials have conducted subgroup analyses based on baseline renal function. These analyses do not establish that minor hemoglobin shifts are independent of underlying diabetic kidney disease.

### 6.2. Special Populations and Clinical Case Reports

Data on hereditary hemochromatosis (HH) demonstrate lower serum ferritin levels in GLP-1 RA users, although an isolated report described acute pancreatitis in a patient with coexisting cystic fibrosis [77,78]. Conversely, in cases of sickle cell anemia—a disease driven by chronic vascular inflammation—preliminary studies indicate that GLP-1 analogs may reduce cardiovascular events, sickle cell crises, and venous thromboembolism [79,80]. A few rare gastrointestinal complications have also been reported. For example, Sekhon et al. documented ischemic colitis in a 32-year-old woman six weeks after initiating tirzepatide for weight loss [81]. Although colonoscopy revealed sigmoid erosions and ulcerations, her hemoglobin levels remained stable with conservative management [81]. In another case, a 32-year-old woman presenting with iron-deficiency anemia was incidentally found to have transient, non-obstructive small-bowel intussusception on CT enterography while taking semaglutide, which resolved without surgical intervention [82]. Thus, while case reports provide valuable signals for detecting rare clinical phenomena, they inherently lack control groups and cannot establish a direct causal relationship.

### 6.3. Dialysis Cohorts and Anti-Inflammatory Effects

Patients with end-stage renal disease (ESRD) on maintenance hemodialysis represent a unique clinical population. In this population, anemia pathogenesis is multifactorial and confounded by chronic uremic inflammation, decreased erythropoietin synthesis, a shortened erythrocyte lifespan, secondary hyperparathyroidism, blood loss, and external therapeutic interventions such as intravenous iron administration and erythropoiesis-stimulating agents (ESAs). Consequently, evaluating the isolated hematological impact of incretin-based therapies in this setting requires careful consideration of these covariates. In a matched cohort of 2468 U.S. adult dialysis patients, Lama et al. observed that 12 months of GLP-1 RA therapy was associated with marked reductions in systemic inflammation and higher serum albumin levels [57]. Although hemoglobin concentrations remained similar between GLP-1 RA users and matched controls (109.0 vs. 108.4 g/L) [57], the potential clinical relevance of GLP-1 RA therapy may lie in its indirect hematological effects. It is critical to emphasize that static or similar hemoglobin levels alone do not demonstrate a direct anti-anemic or erythropoietic effect. Instead, the potential relevance in this cohort is indirect and may be mediated through modulation of inflammatory pathways. Specifically, the GLP-1 RA group required lower cumulative doses of erythropoiesis-stimulating agents (ESA dose-sparing effect) and exhibited a significantly lower erythropoietin resistance index, supported by higher serum ferritin and TSAT levels [57]. Nevertheless, given the observational nature of these data, these findings must be interpreted cautiously. Residual confounding and indication bias cannot be excluded, and stable hemoglobin concentrations in ESRD reflect a complex equilibrium between exogenous drug administration, systemic disease severity, and nutritional status.

### 6.4. Population-Level TriNetX Analysis: Hemoglobin and Anemia Outcomes

Chronic metabolic inflammation suppresses erythropoiesis via interleukin-6 and hepcidin-mediated iron trapping [48,49,50]. To assess whether GLP-1 RAs may improve erythropoiesis by dampening inflammatory cascades, a propensity score-matched analysis of 10,592 adults with T2DM was conducted using the TriNetX federated network (5296 GLP-1 RA initiators vs. 5296 matched controls) [83]. After 12 months, GLP-1 RA users had significantly higher hemoglobin (121.8 vs. 117.8 g/L) and hematocrit levels (36.97% vs. 35.92%) [83], and these differences persisted for 24 months (hemoglobin 123.1 vs. 118.2 g/L; hematocrit 37.31% vs. 36.02%) [83]. These findings represent differences in hemoglobin and hematocrit concentrations between groups and should not be interpreted as evidence of reduced anemia incidence. GLP-1 RA users also exhibited a lower red cell distribution width and a modest decrease in mean corpuscular volume, representing differences in red-cell indices between groups [83]. Among individuals without baseline anemia, GLP-1 RA use was associated with a lower incidence of severe anemia at 24 months, as determined based on predefined hemoglobin thresholds: hemoglobin < 100 g/L: 0.7% (GLP-1 RA) vs. 1.3% (controls) and hemoglobin < 90 g/L: 0.3% (GLP-1 RA) vs. 0.7% (controls) [83]. Because of the observational design, residual confounding and indication bias cannot be excluded.

### 6.5. Discrepancies in the Literature

**Figure 2:**
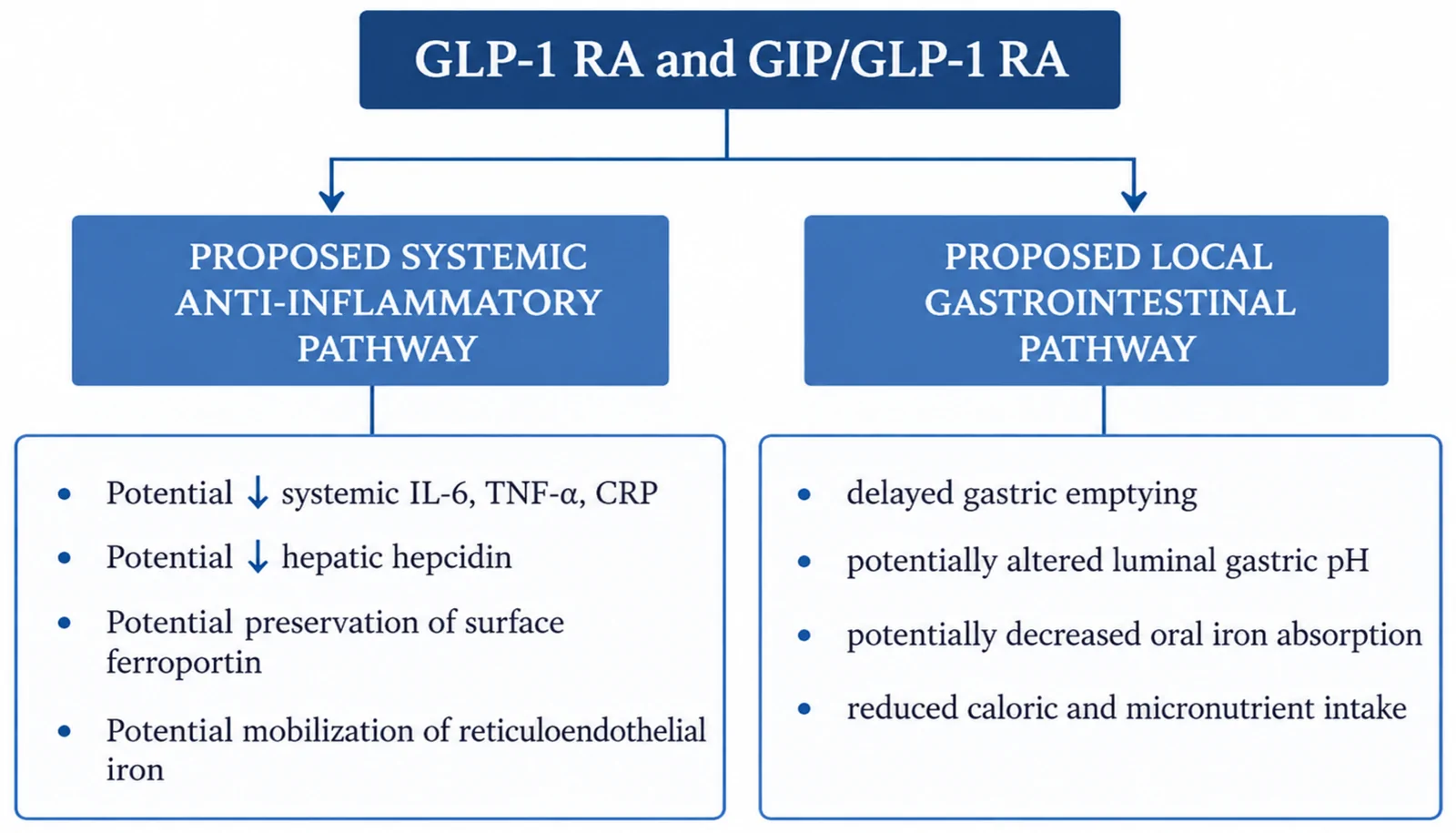
Proposed dual pathways that may influence anemia during GLP-1 RA and GIP/GLP-1 receptor agonist therapy.

The apparent paradox found within the existing literature—where specific investigations associate GLP-1 and combined GLP-1/GIP treatments with incident anemia, whereas others report potential hematoprotective effects—may reflect two competing physiological mechanisms operating in opposite directions. Ultimately, the overall clinical outcome for any specific cohort may reflect the balance between systemic anti-inflammatory effects and potential reductions in dietary iron intake or iron absorption. Within patient groups affected by chronic low-grade renal or metabolic inflammation, such as individuals with T2DM, chronic kidney disease, or those receiving maintenance hemodialysis, anemia is primarily driven by inflammatory signaling cascades [11,12]. Persistent systemic elevations of pro-inflammatory cytokines, together with decreased renal clearance, may contribute to hepcidin accumulation [48,49,50,84]. By mitigating systemic inflammatory pathways and reducing circulating concentrations of pro-inflammatory cytokines, GLP-1/GIP receptor agonists may modulate inflammatory pathways, potentially influencing iron mobilization [51,84]. Large-scale clinical assessments and population database reviews focused on dialysis groups have predominantly captured this potential anti-inflammatory pathway, reporting upward hemoglobin trends, lower red cell distribution width, and decreased requirements for exogenous erythropoiesis-stimulating agents [57,83]. In contrast, observational studies examining outpatient groups initiating incretin therapy primarily for metabolic control or weight management highlight a potential localized gastrointestinal process affecting nutrient absorption [56,74]. Additionally, activation of incretin receptors delays gastric emptying and may modify intraluminal pH, potentially affecting non-heme iron solubilization and subsequent duodenal enterocyte uptake [74]. Simultaneously, potent central appetite suppression, together with prolonged post-meal fullness, may result in significant caloric reductions, decreased overall dietary variety, and lower consumption of essential micronutrients such as riboflavin, vitamin B12, and iron [55,67,68]. Thus, rapid body weight reductions and limitations in food intake may influence downstream systemic iron regulation pathways [53,74]. Moreover, profound energy restriction and rapid adipose tissue turnover may trigger temporary localized metabolic changes that could further influence micronutrient trafficking. Over extended treatment durations, these changes may contribute to reduced iron availability, particularly in individuals with low dietary iron intake or pre-existing nutritional vulnerability (Figure 2) [56,74].

A summary of the main clinical studies evaluating hematological outcomes associated with incretin-based and related glucose-lowering therapies is provided in Table 1.

## 7. The Era of Dual and Triple Agonists

### 7.1. Tirzepatide: Clinical Evidence and Nutritional Considerations

Tirzepatide is a synthetic 39-amino-acid peptide that acts as a dual GIP and GLP-1 receptor agonist and has an elimination half-life of approximately 5 days, supporting once-weekly administration [85]. Broad clinical evaluation has shown that tirzepatide demonstrates non-inferiority—and often superiority—to selective GLP-1 receptor agonists with regard to glycemic control, nephroprotection, and cardiovascular risk reduction while maintaining a similar gastrointestinal safety profile [85,86,87,88,89,90,91,92]. Despite a rapidly growing literature base exceeding 2300 publications, empirical investigation of dual agonism in the context of incident anemia remains exceptionally sparse. In a targeted cross-sectional assessment of 69 individuals undergoing weight-loss therapy with incretin mimetic agents, Johnson et al. evaluated dietary records over a 3-day period to quantify micronutrient density [62]. Their findings highlighted widespread nutritional inadequacies across the cohort, with a prominent deficit in daily dietary iron intake [62].

### 7.2. Mechanistic Considerations Regarding Tirzepatide and Hematological Outcomes

Although tirzepatide has been extensively evaluated in randomized clinical trials, direct clinical evidence specifically addressing its effects on hemoglobin levels remains limited. Major clinical trials have predominantly reported gastrointestinal adverse events, such as nausea, diarrhea, vomiting, and decreased appetite, rather than anemia as a characteristic treatment-related adverse event [85,86,87,88,89,90,91,92]. Several mechanisms could theoretically influence hematological parameters in susceptible individuals. Tirzepatide produces substantial reductions in food intake and body weight, and its gastrointestinal effects may contribute to reduced dietary intake and potentially lower consumption of iron and other micronutrients. However, these pathways should currently be regarded as plausible hypotheses rather than established causes of anemia. Recent post hoc analyses of the SURMOUNT-1 to -4 trials found that treatment-emergent events related to macronutrient malnutrition and vitamin deficiencies were uncommon, while routine measurement of vitamin and mineral concentrations was not performed, limiting conclusions regarding micronutrient status [93]. Conversely, the metabolic and anti-inflammatory effects of tirzepatide could theoretically influence iron homeostasis through pathways involving systemic inflammation and hepcidin regulation. However, direct effects have not been established in humans. Therefore, current evidence does not support classifying tirzepatide as either a hematoprotective treatment or an anemia-inducing therapy. Any potential relationship between tirzepatide treatment and hematological changes is likely to be influenced by baseline anemia and iron status, nutritional intake, gastrointestinal tolerance, kidney function, inflammation, and other patient-specific factors. Thus, prospective studies incorporating predefined hematological and nutritional endpoints are required to determine whether tirzepatide has clinically meaningful effects on anemia or iron metabolism.

### 7.3. Retatrutide: Triple-Receptor Agonism and Metabolic Horizons

Retatrutide is an investigational single-molecule triple agonist targeting the GLP-1, GIP, and glucagon receptors [94]. Phase 2 clinical trials demonstrated substantial dose-dependent reductions in body weight and improvements in glycemic control, with gastrointestinal adverse events representing the most frequently reported side effects [94,95,96,97,98]. Because retatrutide remains investigational, clinical evidence regarding its long-term hematological effects is particularly limited, and no causal association between retatrutide and anemia has been established. The marked effects of retatrutide on appetite and body weight raise the hypothesis that substantial caloric restriction, reduced dietary diversity, or rapid weight loss could indirectly influence micronutrient availability and, consequently, hematological parameters in nutritionally vulnerable individuals. However, this possibility has not been demonstrated clinically and should not be interpreted as evidence that retatrutide causes anemia. Similarly, the metabolic consequences of glucagon receptor co-activation could theoretically influence energy and amino-acid metabolism relevant to erythropoiesis, but direct effects on erythrocyte production or systemic iron homeostasis have not been established in humans. These proposed pathways should therefore be considered mechanistic hypotheses requiring experimental and clinical validation.

### 7.4. Oral Nonpeptide Agonists

Paralleling peptide development, orforglipron—an oral, nonpeptide, small-molecule GLP-1 receptor agonist—is undergoing clinical development for chronic weight management [99,100]. An extended 72-week clinical evaluation in adults with obesity confirmed its superior weight reduction effects relative to placebos, accompanied by a gastrointestinal adverse-event profile consistent with injectable incretins [99,100]. As additional next-generation small molecules and multi-target peptides transition into clinical practice, prospective research must specifically track their potential downstream effects on micronutrient absorption and long-term hematological safety. Thus, retatrutide and orforglipron remain investigational agents in the context discussed here, and any potential hematological or nutritional effects should therefore be considered hypothesis-generating rather than established clinical effects.

## 8. Causal Link vs. Epiphenomenon: Confounding Factors in Clinical Practice and the Incretin Paradox

Modern clinical management of type 2 diabetes and obesity has evolved beyond glycemic control, prioritizing multi-organ protection across cardiovascular, renal, and metabolic domains. Although DPP-4 inhibitors, GLP-1 receptor agonists, and dual GLP-1/GIP agonists all target the incretin system, their clinical and laboratory profiles—particularly regarding hematological parameters and nutritional status—diverge significantly due to fundamental differences in pharmacological potency, receptor engagement, and adverse event profiles. While DPP-4 inhibitors exert a neutral impact on nutrient absorption, potent GLP-1 and GLP-1/GIP receptor agonists alter gastrointestinal physiology and appetite signaling, which may subsequently modify nutrient absorption and iron dynamics. Intriguingly, safety reporting from major clinical trial programs evaluating dual- and mono-incretin therapies—including SURMOUNT-2, SURMOUNT-3, SURPASS-5, and SURMOUNT-CN—did not register anemia as a frequent adverse event, nor did they report clinically relevant declines in baseline hemoglobin [87,101,102,103,104,105].

This dichotomy raises the following critical clinical questions:

Why do pharmacotherapies targeting the same endocrine pathway produce divergent hematological outcomes?

Why do the systemic anti-inflammatory actions of GLP-1/GIP agonists not uniformly counteract malabsorptive iron deficits?

Does greater metabolic and weight-loss efficacy inherently carry a higher risk of hidden nutritional vulnerability?

Why do in vivo and in vitro study results usually not overlap?

It remains to be fully determined whether chronic engagement of incretin receptors exerts direct protective effects on the bone marrow niche by suppressing local microenvironmental inflammation, modulating hepcidin dynamics, or altering hematopoiesis. Additionally, anemia affects approximately 27% of individuals with type 2 diabetes independent of incretin use, with the highest prevalence observed among those with concomitant chronic kidney disease [106]. Decreased hemoglobin concentrations serve as a strong independent predictor of adverse cardiorenal events [107]. Thus, prospective clinical cohorts and translational in vivo and in vitro studies with primary hematological endpoints are required to clarify these molecular mechanisms. Furthermore, while prescriptions for incretin-based therapies have surged globally, clinical integration of structured nutritional guidance has lagged behind. Only a few real-world studies document systematic dietary counseling, track long-term macronutrient shifts, or involve registered dietitians during therapy escalation [108]. As debates continue regarding whether widespread incretin utilization reflects an evidence-based clinical transformation or an overprescribed trend, clinicians must carefully select appropriate candidates [109,110,111,112,113,114,115,116,117]. Routine hematological and micronutrient testing for all patients receiving incretin-based therapy is not currently supported by sufficient evidence. However, individualized assessment may be appropriate in patients with pre-existing anemia, nutritional risk, substantial gastrointestinal intolerance, marked weight loss, chronic kidney disease, or other clinical features suggesting an increased risk of nutritional or hematological abnormalities [118,119,120,121,122,123,124,125].

### Major Potential Confounders of Hematological Outcomes

Interpreting hematological changes during incretin-based therapy requires careful consideration of several potential confounders that may independently influence hemoglobin concentration. Specifically, baseline hematological status is particularly important, as patients entering treatment with pre-existing anemia, depleted iron stores, or lower baseline hemoglobin may have a substantially different subsequent trajectory than those with normal hematological parameters. Higher baseline hemoglobin has been associated with a lower likelihood of incident anemia in observational cohorts [56]. The severity of chronic kidney disease should also be considered because disease- and treatment-related factors may contribute to anemia independently of these medications [106,107]. Systemic inflammation may modify iron availability, potentially counterbalancing hematological effects attributable to incretin therapy [57]. Sex-related differences are also relevant, particularly in premenopausal women, in whom menstrual blood loss may contribute to iron deficiency and should be distinguished from treatment-associated changes. Gastrointestinal blood loss represents another important alternative explanation for iron-deficiency anemia, particularly in patients with CKD or other comorbidities. Thus, dietary iron intake and the use of iron or other micronutrient supplements can modify hematological outcomes. Concomitant medications may represent additional confounders: metformin and proton pump inhibitors are associated with lower vitamin B12 concentrations [126,127]. Finally, the choice of antidiabetic therapy itself may introduce confounding by indication, as treatment selection is influenced by baseline metabolic status, renal function, obesity, comorbidity burden, and concomitant medications. Therefore, observed differences in hemoglobin or anemia incidence between incretin-based therapies and other antidiabetic agents should not automatically be interpreted as drug-specific effects.

## 9. Conclusions and Future Directions

Current evidence does not establish incretin-based therapies as a class cause of anemia. Rather, selected observational and mechanistic findings raise the possibility that hematological outcomes may reflect an interaction between treatment-related changes in food intake, gastrointestinal physiology, iron absorption, weight loss, inflammation, kidney function, and baseline nutritional status. Moreover, evidence directly linking incretin therapy to clinically significant anemia remains limited and heterogeneous. The available clinical literature therefore supports a cautious, individualized interpretation rather than routine attribution of anemia to incretin therapy. Thus, prospective studies incorporating standardized definitions of anemia, iron status, nutritional biomarkers, gastrointestinal symptoms, dietary intake, kidney function, and longitudinal treatment exposure are needed to determine whether specific patient subgroups are at increased risk. In clinical practice, hematological and nutritional assessment should be individualized according to baseline risk factors and treatment-related clinical features rather than applied universally.

## Figures and Tables

**Table 1:** Clinical studies evaluating hematological outcomes associated with incretin-based and related glucose-lowering therapies.

Drug Class/Therapy	Study Population	Study Design	N	Comparator	Duration	Hematological Outcome	Effect Estimate/Main Finding	Main Limitations	Outcome Definition/Parameter Assessed
GLP-1 receptor agonists	Adults with type 2 diabetes	Retrospective observational cohort	461,382	—	6–12 months	Iron-deficiency anemia; non-iron nutritional anemia; nutritional deficiencies	IDA: 1.6% at 6 months and 3.2% at 12 months; non-iron nutritional anemia: 0.5% and 1.1%; overall nutritional deficiencies: 12.7% and 22.4%	Retrospective; no causal inference; nutritional status and other confounders may influence outcomes [53]	Reported iron-deficiency anemia and non-iron nutritional anemia; overall nutritional deficiencies at 6 and 12 months. Diagnostic criteria not specified in the manuscript.
GLP-1 analog therapy	Adults receiving GLP-1 analog treatment	Retrospective study	700	—	≥90 days	Hemoglobin; incident anemia; ferritin	Median Hb decrease: 2 g/L; incident anemia: 8.4%; ferritin largely unchanged	Retrospective; no prospective confirmation; potential residual confounding [56]	Change in hemoglobin concentration; incident anemia; ferritin. Anemia definition not specified in the manuscript.
Semaglutide	Patients with type 2 diabetes mellitus	Pilot study/oral iron absorption assessment	51	Within-patient pre-treatment response	10 weeks	Serum iron; TSAT; ferritin; iron absorption	Serum iron response 19% → 8%, TSAT 17% → 7%, ferritin 3% → 2%; median iron uptake reduction 13%; 17.6% had >30% reduction	Small sample; short follow-up; exploratory; does not establish clinically significant iron deficiency or anemia [74]	Post-oral iron serum iron response; transferrin saturation (TSAT); ferritin; estimated iron absorption/uptake.
GLP-1 receptor agonists	Adults with T2DM	Propensity-score-matched real-world cohort (TriNetX)	10,592 (5296 vs. 5296)	Matched controls	12 and 24 months	Hb; Hct; RDW; MCV; severe anemia thresholds	12 months Hb 121.8 vs. 117.8 g/L; Hct 36.97% vs. 35.92%. 24 months Hb 123.1 vs. 118.2 g/L; Hct 37.31% vs. 36.02%. Severe anemia at 24 months: Hb < 100 g/L, 0.7% vs. 1.3%; Hb < 90 g/L, 0.3% vs. 0.7%	Observational; Hb/Hct differences should not be interpreted as reduced overall anemia incidence; residual confounding possible [83]	Hemoglobin and hematocrit changes; RDW and MCV; severe anemia defined by Hb < 100 g/L and Hb < 90 g/L.
GLP-1 receptor agonists	Adults with ESRD on maintenance hemodialysis	Matched observational cohort	2468	Matched controls	12 months	Hb; inflammation; albumin; ESA requirement/EPO resistance	Hb 109.0 vs. 108.4 g/L; reduced inflammation, higher albumin, and lower ESA requirement/EPO resistance	Selected population; anemia confounded by ESRD, dialysis care, iron and ESA treatment; stable Hb does not demonstrate direct erythropoiesis [57]	Hemoglobin; inflammatory markers; albumin; ESA requirement and erythropoietin resistance.
SGLT2 inhibitors vs. GLP-1 RAs	Adults with T2D and CKD stages 1–3	Multicenter retrospective cohort	13,799	GLP-1 receptor agonists	2.5 years	Composite anemia events	Fewer composite anemia events with SGLT2 inhibitors than with GLP-1 RAs	Retrospective comparative design; class differences and baseline characteristics may confound association [75]	Composite anemia events.
SGLT2i vs. GLP-1 RA vs. DPP-4i	Patients initiating glucose-lowering therapy	Danish population-based active-comparator new-user cohort	132,914	Active comparators	NR	Anemia; secondary polycythemia	DPP-4i initiation associated with higher anemia risk than SGLT2i and GLP-1 RA; SGLT2i had more secondary polycythemia	Observational; treatment selection and residual confounding; association does not establish class-wide causality [76]	Anemia incidence; secondary polycythemia.
DPP-4 inhibitors	Hemodialysis patients without iron deficiency	Observational cohort	NR	NR	NR	Erythropoietin resistance index	Improvement in erythropoietin resistance index among patients without iron deficiency	Selected dialysis population; observational; exact N/duration not specified in manuscript [29]	Erythropoietin resistance index.
DPP-4 inhibitors	Diabetic kidney disease	Retrospective before–after cohort	443	Pre-treatment period	NR	Hemoglobin	Hb 119.8 ± 20.7 → 118.7 ± 21.2 g/L; decline smaller than pre-treatment decline	Small absolute change; retrospective; no causal inference [23]	Hemoglobin concentration/change from pre-treatment period.
DPP-4 inhibitor + metformin	Adults with diabetes without major confounding comorbidities	Comparative observational study	110	Metformin alone	8 months	Hemoglobin; MCV	No significant difference in Hb or MCV between groups	Small sample; short follow-up; limited generalizability [30]	Hemoglobin concentration and MCV; between-group comparison.
DPP-4 inhibitors vs. SGLT2 inhibitors	Patients with T2DM	Cohort study	28,441	SGLT2 inhibitors	5 years	Anemia incidence	5-year cumulative incidence: 11.3% with DPP-4i vs. 6.9% with SGLT2i	Observational; residual confounding; distinguish general anemia from IDA unless iron deficiency was specifically documented [31]	Incident anemia over 5 years.
Sitagliptin	Patients undergoing hematopoietic stem cell transplantation	Randomized phase II clinical trial	NR	NR	NR	Graft-versus-host disease (primary outcome)	Evaluated sitagliptin for prevention of acute GVHD	Primary outcome was GVHD rather than anemia/Hb; does not establish a hematopoietic effect [33]	Primary outcome: acute graft-versus-host disease; hematological anemia/Hb outcomes were not directly assessed.

Abbreviations: CKD, chronic kidney disease; DPP-4i, dipeptidyl peptidase-4 inhibitor; EPO, erythropoietin; ESA, erythropoiesis-stimulating agent; GLP-1 RA, glucagon-like peptide-1 receptor agonist; Hb, hemoglobin; Hct, hematocrit; IDA, iron-deficiency anemia; MCV, mean corpuscular volume; NR, not reported; RDW, red cell distribution width; SGLT2i, sodium–glucose cotransporter-2 inhibitor; T2DM, type 2 diabetes mellitus; TSAT, transferrin saturation.

## Data Availability

No new data were created or analyzed in this study. Data sharing is not applicable to this article.

## Abbreviations

The following abbreviations are used in this manuscript:

DPP-4	Dipeptidyl peptidase-4
DPP-4i	Dipeptidyl peptidase-4 inhibitor
GLP-1	Glucagon-like peptide-1
GLP-1 RA	Glucagon-like peptide-1 receptor agonist
GIP	Glucose-dependent insulinotropic peptide
T2DM	Type 2 diabetes mellitus
SGLT2i	Sodium–glucose cotransporter-2 inhibitor
BM	Bone marrow
FDA	Food and Drug Administration
EMA	European Medicines Agency
OIAT	Oral iron absorption test
HH	Hereditary hemochromatosis
MCV	Mean corpuscular volume
HIF-1α	Hypoxia-inducible factor 1-alpha
SDF-1	Stromal cell-derived factor 1
IL-6	Interleukin-6
ESA	Erythropoiesis-Stimulating Agent
ESRD	End-stage renal disease
eGFR	estimated glomerular filtration rate
vs.	versus
